# BH3 mimetics in combination with nilotinib or ponatinib represent a promising therapeutic strategy in blast phase chronic myeloid leukemia

**DOI:** 10.1038/s41420-022-01211-1

**Published:** 2022-11-15

**Authors:** Narissa Parry, Caroline Busch, Victoria Aßmann, Jennifer Cassels, Alan Hair, G. Vignir Helgason, Helen Wheadon, Mhairi Copland

**Affiliations:** 1 Paul O’Gorman Leukaemia Research Centre, School of Cancer Sciences, University of Glasgow, Glasgow, UK; 2 Wolfson Wohl Cancer Research Centre, School of Cancer Sciences, University of Glasgow, Glasgow, UK

**Keywords:** Chronic myeloid leukaemia, Apoptosis

## Abstract

Dysregulation of the BCL-2 family is implicated in protecting chronic myeloid leukemia (CML) cells from intracellular damage and BCR::ABL1-inhibition with tyrosine kinase inhibitors (TKIs) and may be a viable therapeutic target in blast phase (BP-)CML, for which treatment options are limited. BH3 mimetics, a class of small molecule inhibitors with high-specificity against the prosurvival members of the BCL-2 family, have displayed clinical promise in the treatment of chronic lymphocytic and acute myeloid leukemia as single agents and in combination with standard-of-care therapies. Here we present the first comparison of inhibition of BCL-2 prosurvival proteins BCL-2, BCL-xL and MCL-1 in combination with a second or third generation TKI, crucially with comparisons drawn between myeloid and lymphoid BP-CML samples. Co-treatment of four BP-CML cell lines with the TKIs nilotinib or ponatinib and either BCL-2 (venetoclax), MCL-1 (S63845) or BCL-xL (A-1331852) inhibitors resulted in a synergistic reduction in cell viability and increase in phosphatidylserine (PS) presentation. Nilotinib with BH3 mimetic combinations in myeloid BP-CML patient samples triggered increased induction of apoptosis over nilotinib alone, and a reduction in colony-forming capacity and CD34^+^ fraction, while this was not the case for lymphoid BP-CML samples tested. While some heterogeneity in apoptotic response was observed between cell lines and BP-CML patient samples, the combination of BCL-xL and BCR::ABL1 inhibition was consistently effective in inducing substantial apoptosis. Further, while BH3 mimetics showed little efficacy as single agents, dual-inhibition of BCL-2 prosurvival proteins dramatically induced apoptosis in all cell lines tested and in myeloid BP-CML patient samples compared to healthy donor samples. Gene expression and protein level analysis suggests a protective upregulation of alternative BCL-2 prosurvival proteins in response to BH3 mimetic single-treatment in BP-CML. Our results suggest that BH3 mimetics represent an interesting avenue for further exploration in myeloid BP-CML, for which alternative treatment options are desperately sought.

## Introduction

Chronic myeloid leukemia (CML) is a triphasic disease consisting of chronic, accelerated, and blast phases (CP-, AP-, and BP-CML, respectively). BP-CML can be further classified as myeloid, lymphoid, or biphenotypic, depending on morphology and surface markers present on the blast cells [1]. CML is driven by BCR::ABL1 [2], which is targetable with tyrosine kinase inhibitors (TKIs), the first of which was imatinib [3]. Second and third generation (2G and 3G) TKIs have since been developed, including dasatinib [4], nilotinib [5], bosutinib [6] and ponatinib [7], which are used for patients who have failed imatinib, including progression to BP-CML [8, 9]. However, despite these advances, treatment with TKI is often ineffective due to the distinct molecular profiles associated with BP-CML [10]. Unfortunately, the outcomes for patients with BP-CML have not improved, with life expectancies of less than a year [9]; end-stage disease therefore represents a significant unmet challenge in CML.

Apoptosis is a form of programmed cell death controlled by the balance of the BCL-2 family of proteins [11]. These proteins share common BCL-2 homology (BH) domains, and can be categorized as prosurvival (e.g., BCL-2, MCL-1, BCL-xL) or proapoptotic (e.g., BIM, BAX, BAK) [12, 13]. During apoptosis, BAX and BAK form pores in the mitochondrial outer membrane, which releases proapoptotic factors such as cytochrome c and initiating apoptosis through the formation of the apoptosome and activation of executioner caspases [14, 15].

BCR::ABL1 confers a survival advantage to CML cells in part through changes to the BCL-2 proteins [16], particularly through upregulation of BCL-2 [17, 18], MCL-1 [19], and BCL-xL [20, 21], and downregulation of BIM [22–24]. Further, BCL-xL has been implicated in CML progression to BP [25], and BCL-2 and BCL-xL dependence are associated with a more immature immune cell phenotype [26, 27]. Small molecule inhibitors of the prosurvival BCL-2 proteins, termed ‘BH3 mimetics’, are potent inducers of apoptosis in cells with imbalances in the BCL-2 family [28]. The best studied BH3 mimetic, venetoclax (VEN; formerly ABT-199), has been approved by the U.S. Food and Drug Administration in combination with rituximab for treatment-naïve chronic lymphocytic leukemia with a 17p deletion [29] and in acute myeloid leukemia with hypomethylating agents or low-dose cytarabine for cases in which intensive chemotherapy is not an option [30, 31].

Although prosurvival shifts within the BCL-2 family have been reported in CML and a number of pre-clinical combinations of TKIs with BH3 mimetics have been carried out and display promise [16], comparisons of BH3 mimetic responses within BP-CML are few. In this study, three BH3 mimetics were chosen for further investigation; the BCL-2 inhibitor venetoclax [32], the MCL-1 inhibitor S63845 [33], and the BCL-xL inhibitor A-1331852 [34].

## Results

### BCL-2 prosurvival protein inhibition reduces cell viability and induces apoptosis in BP-CML cell lines

**Fig. 1:**
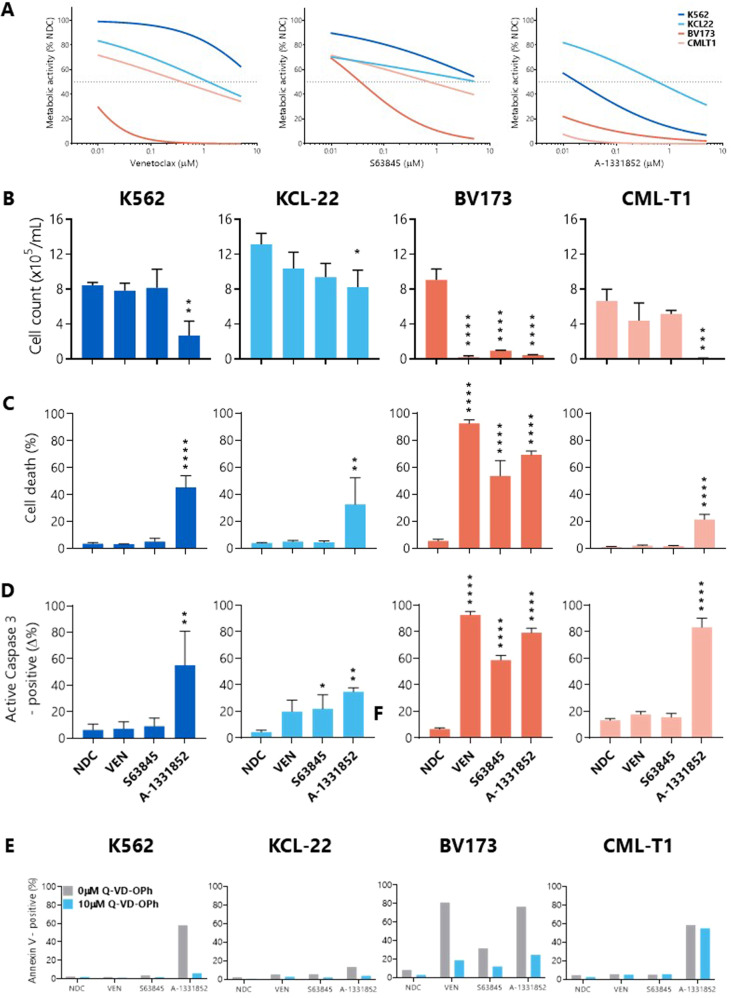
BCL-xL inhibition reduces cell viability and induces apoptosis in BP-CML cell lines. **A** BP-CML cell lines were treated for 72 h with increasing concentrations of venetoclax (VEN; ABT-199), S63845, or A-1331852 before incubation with resazurin. Lines of best fit for means of replicates, *N* = 3, dotted line indicates 50% inhibition. **B** Cell lines were treated with 1 µM of each indicated BH3 mimetic for 72 h before viable cell counts, Annexin V/DAPI staining (**C**), and active caspase-3 staining (**D**) were measured (*N* = 3). **E** BP-CML cell lines were pre-treated with the pan-caspase inhibitor Q-VD-OPh (10 µM) before 1 µM BH3 mimetic treatment for 72 h and subsequent staining with Annexin V, *N* = 2. NDC, no drug control. One-way ANOVA, *p ≤ 0.05, **p ≤ 0.01, ***p ≤ 0.001, ****p ≤ 0.0001. Data are represented as mean ± SD.

IC50 values were determined for BH3 mimetic drugs in four BP-CML cell lines; negligible cell killing was demonstrated at 24 h and 48 h (data not shown), but sensitivity to the drugs was observed after 72 h treatment (Fig. 1A). The most potent BH3 mimetic tested was the BCL-xL-targeting A-1331852 (K562: 0.0188 µM; KCL-22: 0.6 µM; BV173: 0.005 µM; CML-T1: 0.001 µM), although the two lymphoid cell lines were sensitive to all three BH3 mimetics (BV173 VEN: 0.0045 µM, S63845: 0.036 µM; CML-T1 VEN: 0.4 µM, S63845: 0.7 µM).

Broadly, the reductions in viable cell counts (Fig. 1B) and the apoptotic responses (Fig. 1C–E) to the BH3 mimetics corresponded with the resazurin reduction assay data. However, apoptotic responses in CML-T1 were lower than expected, potentially due to the late time point at which this was measured, though this may suggest that the BH3 mimetics do not exclusively induce apoptosis in these cells.

To confirm cell death by apoptosis, the pan-caspase inhibitor Q-VD-OPh was used. As expected, pre-treatment with Q-VD-OPh decreased PS presentation in K562, KCL-22, and BV173 cells in response to BH3 mimetics (Fig. 1E). Interestingly, caspase inhibition was insufficient to inhibit PS presentation in CML-T1 cells when treated with A-1331852, suggesting that other cell death pathways may be activated in response to this treatment, or that caspase inhibition by Q-VD-OPh was insufficient to prevent cell death over a sustained period.

BH3 mimetics therefore induce apoptosis to varying degrees as single agents in BP-CML cell lines.

### BCL-xL inhibition in BP-CML cell lines induces upregulation of BCL-2 and MCL-1

Cells can circumvent apoptosis in response to BH3 mimetic single agent treatment through the upregulation of other prosurvival BCL-2 proteins.

**Fig. 2:**
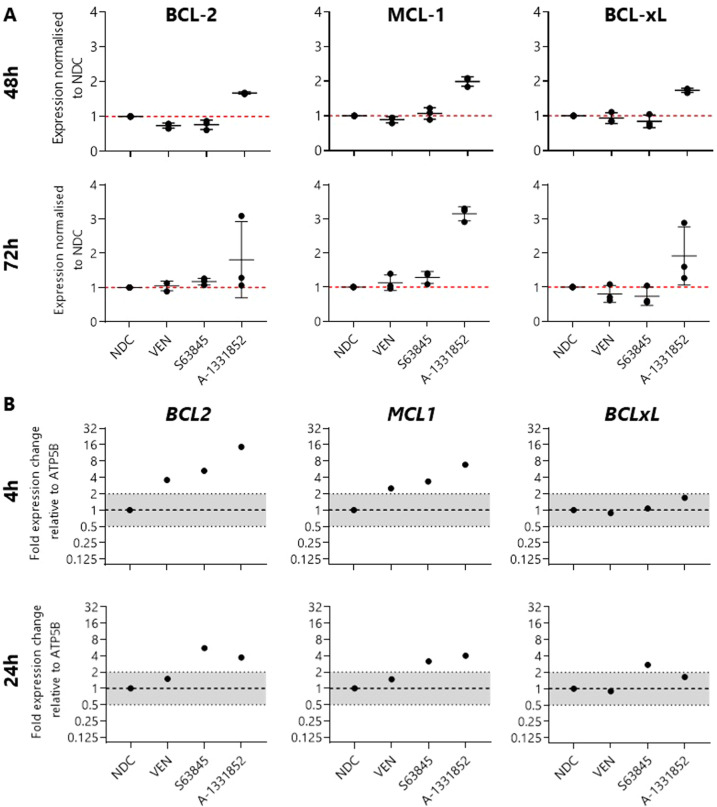
BCL-xL inhibition induces upregulation of BCL-2, MCL-1, and BCL-xL in K562 cells. **A** Protein levels in K562 cells were quantified using median fluorescence intensity and normalized to the no drug control (NDC) after treatment with 1 µM indicated BH3 mimetic after 48 h or 72 h, *N* = 3. (**B**) Gene expression of *BCL2*, *MCL1*, and *BCLxL* in K562 cells treated with 1 µM indicated BH3 mimetics for 4 h or 24 h, *N* = 1. Data are represented as mean ± SD.

To test this in BP-CML cells, K562 cells were treated with 1 µM BH3 mimetics and protein levels were measured using intracellular staining by flow cytometry after 48 h and 72 h treatment. Antibody staining was compared with isotype controls to confirm minimal off-target staining (Supplementary Fig. 1). As shown, treatment of K562 cells with VEN or S63845 does not induce apoptosis, and this does not appear to be due to compensatory upregulation of BCL-2, MCL-1, or BCL-xL (Fig. 2A).

K562 cells are most sensitive to BCL-xL inhibition; strikingly, treatment with A-1331852 resulted in the upregulation of BCL-2 (48 h: 1.68x ± 0.03, 72 h: 1.81x ± 1.11), MCL-1 (48 h: 1.99x ± 0.13, 72 h: 3.15x ± 0.21), and BCL-xL (24 h: 1.74x ± 0.06, 72 h: 1.91x ± 0.85) at the protein level (Fig. 2A), and upregulation of *BCL2* (4 h: 14.60x, 24 h: 3.72x) and *MCL1* (4 h: 6.85x, 24 h: 4.03x) gene expression (Fig. 2B), perhaps as a survival mechanism.

These data suggest a co-dependence on the prosurvival BCL-2 family proteins within BP-CML cell models and provided the rationale for combining BH3 mimetics.

### BCL-2 prosurvival protein combination inhibition results in greater apoptosis induction than single treatments

To observe the additive or synergistic effects of the drugs more clearly, the concentration of BH3 mimetics was reduced to 250 nM. Due to the reliance of cardiac cells on MCL-1 [35] and platelets on BCL-xL [36], decreasing the concentrations of these drugs is also of clinical relevance.

**Fig. 3:**
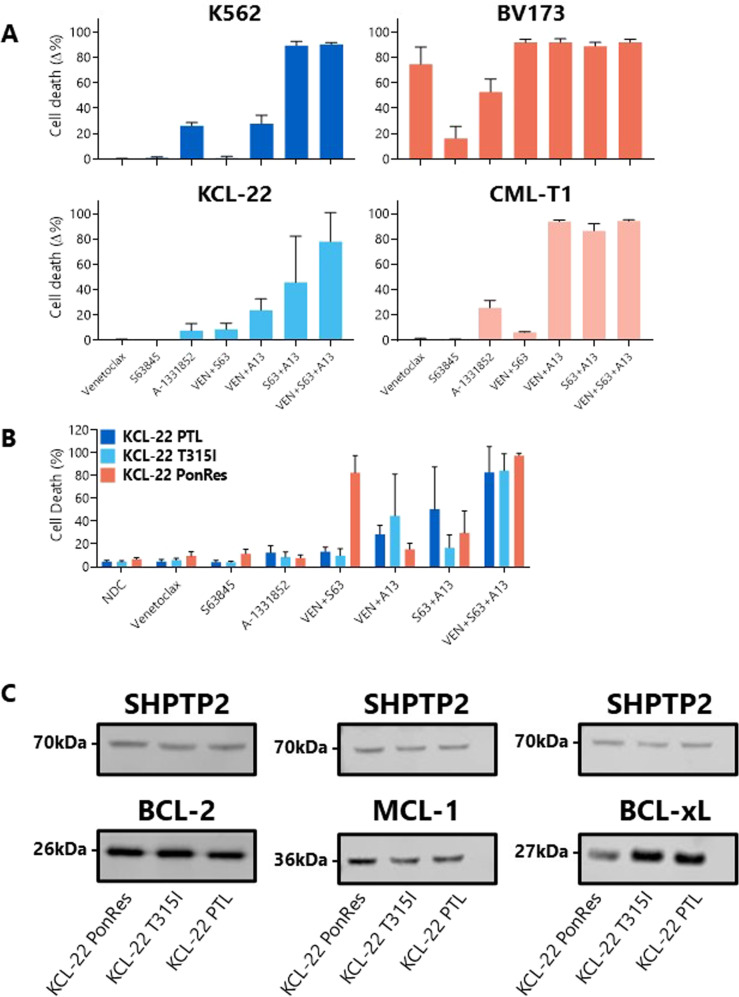
Combining BH3 mimetics in BP-CML cell lines induces a greater degree of apoptosis than single treatments. Parental (**A** analyzed by one-way ANOVA) and TKI-resistant (**B** two-way ANOVA) BP-CML cell lines were treated with 250 nM indicated BH3 mimetic for 72 h before Annexin V/DAPI staining by flow cytometry, *N* = 3. Significant differences can be seen in Supplementary Tables 4 and 5. Basal protein expression of BCL-2, MCL-1, and BCL-xL were measured by Western blot (**C**), with a representative blot for each protein shown, *N* = 3. Full blots in Supplementary Fig. 2. PonRes ponatinib-resistant, PTL parental. Data are represented as mean ± SD.

A potent combination in all cell lines tested was MCL-1 with BCL-xL inhibition, with over 80% cell death observed in K562 (89.44 ± 3.15%), BV173 (88.89 ± 2.91%), and CML-T1 (86.49 ± 5.65%) cells, and over 45% cell death in KCL-22 cells; significantly more than either treatment alone (Fig. 3A, Supplementary Table 4). This suggests that MCL-1 may be used as a secondary survival mechanism against apoptosis upon BCL-xL inhibition in these cells.

The emergence of therapy-resistant disease after treatment with TKIs is one of the biggest challenges faced in the management of CML. As increased dependence on the BCL-2 family has been linked to disease progression and TKI resistance, it was hypothesized that BH3 mimetics may be promising candidates for the treatment of TKI-resistant CML.

To test this, two TKI-resistant cell lines derived from the myeloid BP-CML cell line KCL-22 were identified; the first was developed after continual sublethal exposure to nilotinib and carries the *BCR::ABL1* mutation T315I (KCL-22 T315I) [37], while the second was established after continual sublethal exposure to ponatinib (KCL-22 PonRes) [38] and developed resistance without *BCR::ABL1* kinase mutation. Combinations of the BH3 mimetics were tested on these cell lines and compared with parental KCL-22 cells (Fig. 3B).

Minimal apoptotic responses were observed after single agent treatment with the BH3 mimetics in the TKI-resistant cell lines. However, all cell lines were responsive to combination treatments, with parental KCL-22 cells found to be most sensitive to MCL-1 with BCL-xL inhibition (+45.72% cell death over untreated; *p* = 0.0436), KCL-22 T315I cells to BCL-2 with BCL-xL inhibition (+40.53%; *p* = 0.0576) and KCL-22 PonRes cells to BCL-2 with MCL-1 inhibition (+75.78%; *p* < 0.0001) (Fig. 3B, Supplementary Table 5).

Importantly, the only difference between these cell lines is the previous exposure to either a 2G- or 3GTKI, and the implication is that nilotinib and ponatinib can affect the shift in BCL-2 family dependence in different ways.

To determine whether the basal protein expression levels of BCL-2, MCL-1 and BCL-xL in these cell lines were indicative of apoptotic response after BH3 mimetic treatment, Western blot analyses were carried out (Fig. 3C, Supplementary Fig. 2). The most obvious difference in pro-survival protein expression was the upregulation of MCL-1 (1.75-fold) and the downregulation of BCL-xL (2.17-fold) in the KCL-22 PonRes cell line when normalized to parental KCL-22 values, which may account for the greater sensitivity of these cells to BCL-2/MCL-1 dual inhibition compared with the PTL line. Protein levels alone, however, reveal little about the functional capacity of the pro-survival BCL-2 proteins, and further investigation into the effects of TKIs in these cells would be of interest.

### BH3 mimetics alone and in combination induce a greater degree of apoptosis in BP-CML patient samples than healthy donor samples

For a more clinically relevant application, three healthy donor samples and three myeloid BP-CML patient samples were treated with the BH3 mimetics described above.

**Fig. 4:**
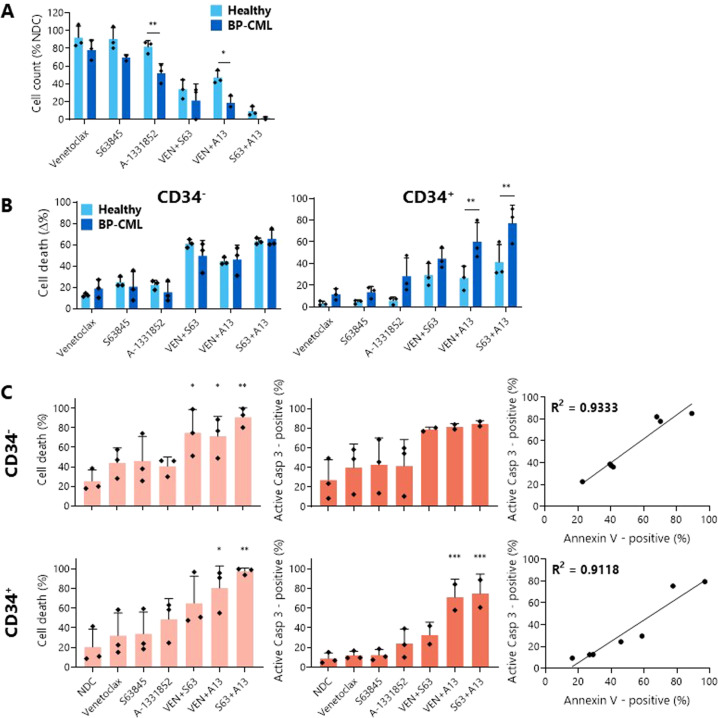
Combining BH3 mimetics in BP-CML patient samples induces a greater degree of apoptosis than single treatments. The effect of 1 µM indicated BH3 mimetic after 72 h treatment in three healthy donor control and three myeloid BP-CML patient samples on viable cell counts (**A**) and Annexin V/DAPI staining (**B**) was measured and analyzed by two-way ANOVA. Significant differences can be seen in Supplementary Tables 6 and 7. Staining with anti-CD34 allowed discrimination between cell death in CD34^−^ and CD34^+^ cells. Apoptosis was confirmed by staining for Annexin V/DAPI (light orange) and active caspase-3 (dark orange) in CD34^−^ and CD34^+^ myeloid BP-CML cells after 72 h treatment with 1 µM BH3 mimetics and correlation between both was determined, *N* = 3 (**C** analyzed by one-way ANOVA). *p ≤ 0.05, **p ≤ 0.01, ***p ≤ 0.001. Data are represented as mean ± SD.

As with the BP-CML cell lines, combinations of BH3 mimetics were more effective at reducing viable cell counts than single agents (Fig. 4A), with S63845 with A-1331852 being the most potent combination (healthy: 9.20 ± 5.30% viable cells; BP-CML: 1.56 ± 1.35%). Notably, BCL-xL inhibition both alone (healthy: 81.72 ± 6.86%; BP-CML: 52.07 ± 10.58%) and with BCL-2 inhibition (healthy: 47.14 ± 7.61%; BP-CML: 18.63 ± 7.51%) significantly reduced the cell counts of BP-CML samples to a greater extent than those of the healthy controls (p = 0.0086, *p* = 0.0121 respectively), indicating a potential therapeutic window.

PS presentation, cell membrane permeabilization, and the presence of active caspase-3 were measured in both the more mature CD34^-^ and primitive CD34^+^ populations to confirm that the decreases in cell counts were due to an increase in apoptosis initiation (Fig. 4B, C). In the healthy and BP-CML samples, the apoptotic responses to the BH3 mimetics within the CD34^-^ fraction were comparable. Within the CD34^+^ population, however, the BP-CML cells were significantly more sensitive than the healthy CD34^+^ cells to BCL-xL inhibition in combination with either BCL-2 (healthy: 26.58 ± 11.02% cell death; BP-CML: 60.20 ± 17.55%; *p* = 0.0077) or MCL-1 (healthy: 41.32 ± 16.19%; BP-CML: 77.09 ± 16.78%; *p* = 0.0043).

### BH3 mimetics selectively kill primitive CD34^+^ BP-CML cells resulting in reduced colony forming capacity

Having determined that BH3 mimetics induce apoptosis to a greater degree in BP-CML CD34^+^ population, which contains disease-driving leukemia stem cells, we investigated whether BH3 mimetics are able to decrease the functional colony-forming capacity of BP-CML cells.

**Fig. 5:**
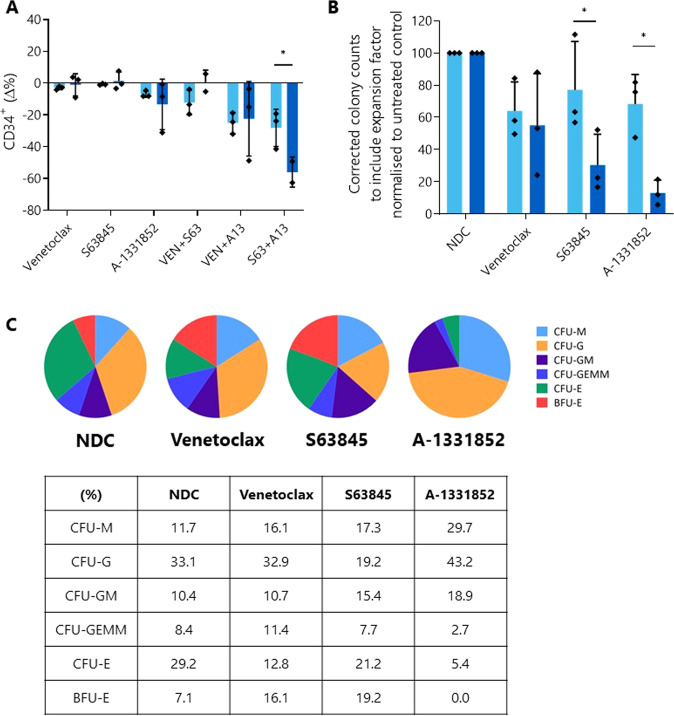
BH3 mimetic treatment reduces the number of primitive cells and the colony-forming capacity of myeloid BP-CML patient samples to a greater degree than healthy samples. **A** Three healthy (light blue) and three myeloid BP-CML patient (dark blue) samples were treated with 1 µM indicated BH3 mimetic for 72 h and the effect on overall percentage CD34^+^ fraction was measured and expressed as a change from the no drug control. **B** Cells from three healthy (light blue) and three BP-CML (two lymphoid, one myeloid; dark blue) samples pre-treated with 1 µM BH3 mimetics for 72 h were plated in Methocult™ and colonies enumerated and normalized to the no drug control (NDC). **C** Representative distribution of colony types (lymphoid BP-CML sample) can be seen as a percentage of total for each arm. Analyzed by two-way ANOVA, *p ≤ 0.05. Data are represented as mean ± SD.

Broadly, the effect of the BH3 mimetics was similar between the healthy and BP-CML samples (Fig. 5A), although when expressed as a change from the no drug control (NDC), the combination of S63845 and A-1331852 reduced the CD34^+^ population in the BP-CML samples to a significantly greater degree than in the healthy samples (healthy: −28.15 ± 11.74%; BP-CML: −55.95 ± 9.48%; *p* = 0.0482), suggesting a specific response to these drugs in the primitive BP-CML cells.

Thereafter, we investigated whether this reduction translated into a functional reduction in the ability of these cells to form colonies in culture as a marker of stem cell potential. After normalization of colony counts to the NDC, a significant difference was seen between the healthy and BP-CML samples after MCL-1 (healthy: 77.24 ± 29.85%; BP-CML: 30.49 ± 19.09%; *p* = 0.0367) or BCL-xL (healthy: 68.32 ± 18.32%; BP-CML: 13.06 ± 7.88; *p* = 0.012) inhibition, suggesting inhibition of these proteins reduces the ability of BP-CML cells to generate colonies to a greater degree than healthy cells (Fig. 5B).

Finally, while the distribution of BP-CML colony types was not affected by VEN or S63845 treatment, this was not the case for cells treated with A-1331852, indicating that BCL-xL inhibition restricts different hematopoietic lineages in vitro (Fig. 5C). After treatment with A-1331852, generally no burst-forming unit-erythroid (BFU-E) colonies grew, though more mature colony-forming unit-erythroid (CFU-E) colonies were seen, albeit at a lower number than for other BH3 mimetics. This corresponds with findings described in the literature of the requirement of BCL-xL in erythropoiesis [39]. Along with the crucial role of BCL-xL in the development of platelets [36, 40, 41], this is an important consideration in the translation of BCL-xL inhibitors to cancer therapy with implications for the toxicity profiles of the drugs, although it should be noted that BP-CML blast cells in patients remain undifferentiated.

### BH3 mimetics in combination with TKIs synergistically reduce cell viability and induce apoptosis in BP-CML cell lines

The aim of BP-CML treatment is to rapidly induce a second CP through the reduction of blast cells and *BCR::ABL1* transcripts in the blood. BH3 mimetics represent a promising therapy to combine with TKIs to achieve this goal.

**Fig. 6:**
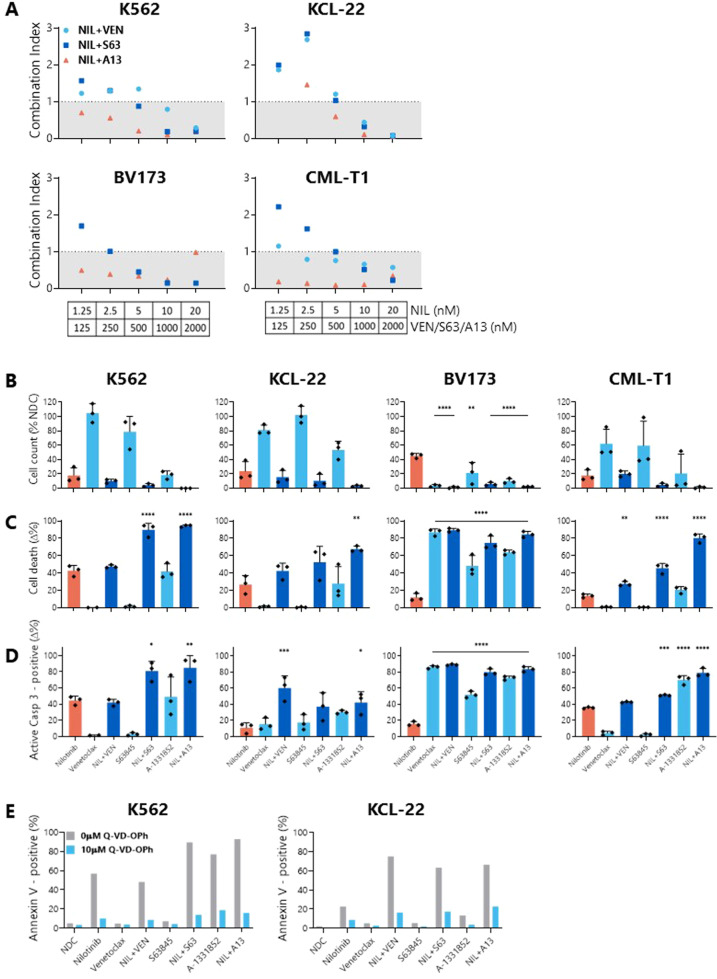
Combinations of BH3 mimetics with nilotinib synergistically reduce cell viability, decrease cell count, and increase apoptosis in BP-CML cell lines over nilotinib alone. **A** BP-CML cell lines were treated with indicated concentrations of nilotinib with either venetoclax (VEN; ABT-199), S63845, or A-1331852 for 72 h before addition of resazurin, *N* = 3. Reductions in cell viability were compared with either nilotinib or BH3 mimetics alone and a combination index (CI) was calculated, as previously described [37]. Values <1 indicate synergistic and >1 indicate antagonistic interactions. Cell lines were treated with 10 nM nilotinib and/or 1 µM indicated BH3 mimetic for 72 h before viable cell counts (**B**), Annexin V/DAPI staining (**C**), or active caspase-3 staining (**D**) was measured. Responses were compared with nilotinib alone, *N* = 3. **E** K562 and KCL-22 cells were pre-treated with 10 µM Q-VD-OPh before treatment with either 10 nM nilotinib and/or 1 µM BH3 mimetics as indicated for 72 h, and the effect on Annexin V/DAPI staining was compared with no Q-VD-OPh treatment. Analyzed by one-way ANOVA, *p ≤ 0.05, **p ≤ 0.01, ***p ≤ 0.001, ****p ≤ 0.0001. Data are represented as mean ± SD.

**Fig. 7:**
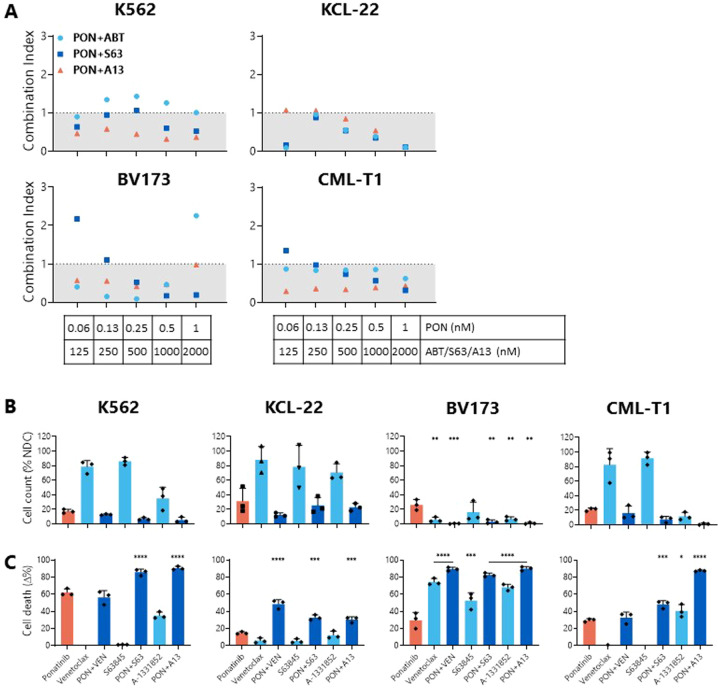
Combinations of BH3 mimetics with ponatinib synergistically reduce cell viability, decrease cell count, and increase apoptosis in BP-CML cell lines over ponatinib alone. **A** BP-CML cell lines were treated with indicated concentrations of ponatinib with either venetoclax (VEN; ABT-199), S63845, or A-1331852 for 72 h before addition of resazurin, *N* = 3. Reductions in cell viability were compared with either ponatinib or BH3 mimetics alone and a combination index (CI) was calculated, as previously described [37]. Values <1 indicate synergistic and >1 indicate antagonistic interactions. Cell lines were treated with 1 nM ponatinib and/or 1 µM indicated BH3 mimetic for 72 h before viable cell counts (**B**) and Annexin V/DAPI staining (**C**) were measured. Responses were compared with ponatinib alone, *N* = 3. PON ponatinib, VEN venetoclax, S63 S63845, A13 A-1331852. Analyzed by one-way ANOVA, *p ≤ 0.05, **p ≤ 0.01, ***p ≤ 0.001, ****p ≤ 0.0001. Data are represented as mean ± SD.

Combination indices for the TKIs nilotinib and ponatinib with VEN, S63845, or A-1331852 were determined after 72 h treatment (Figs. 6A and 7A) and due to the synergistic reductions in cell viability at these concentrations, 1 µM BH3 mimetics with either 10 nM nilotinib or 1 nM ponatinib (chosen after 0.5 nM ponatinib was shown to result in low levels of apoptosis as a single agent) were taken forward for future investigations.

When compared with TKI treatment alone, almost without exception, all cell lines tested demonstrated a reduction in cell count (Figs. 6B and 7B) and significantly more apoptosis (Figs. 6C, D and 7C) after combining either TKI with MCL-1 or BCL-xL inhibition. Combinations of nilotinib with VEN resulted in significantly more PS presentation than nilotinib alone in the lymphoid BP-CML cell lines BV173 (nilotinib: 12 ± 4.46%; nilotinib+VEN: 89.49 ± 2.09%; *p* < 0.0001) and CML-T1 (nilotinib: 13.69 ± 2.05%; nilotinib + VEN: 27.49 ± 2.76%; *p* = 0.0023) (Fig. 6C), and a greater degree of caspase-3 activation in KCL-22 (nilotinib: 10.92 ± 6.23%; nilotinib + VEN: 60.27 ± 15.01%; *p* = 0.0006) and BV173 cells (nilotinib: 15.84 ± 2.83%; nilotinib+VEN: 89 ± 0.83%; *p* = 0.0006) (Fig. 6D). Similarly, combinations of ponatinib with VEN induced significantly greater PS presentation in KCL-22 (ponatinib: 14.73 ± 1.38%; ponatinib+VEN: 48.57 ± 5.04%; *p* < 0.0001) and BV173 cells (ponatinib: 29.70 ± 9.1%; ponatinib + VEN: 89.37 ± 2.15%; *p* < 0.0001) (Fig. 7C). Pan-caspase inhibition with Q-VD-OPh confirmed cell death by apoptosis in K562 and KCL22 cell lines (Fig. 6E).

### Combinations of BH3 mimetics with nilotinib induce significantly more apoptosis in myeloid BP-CML patient samples than healthy donor samples

**Fig. 8:**
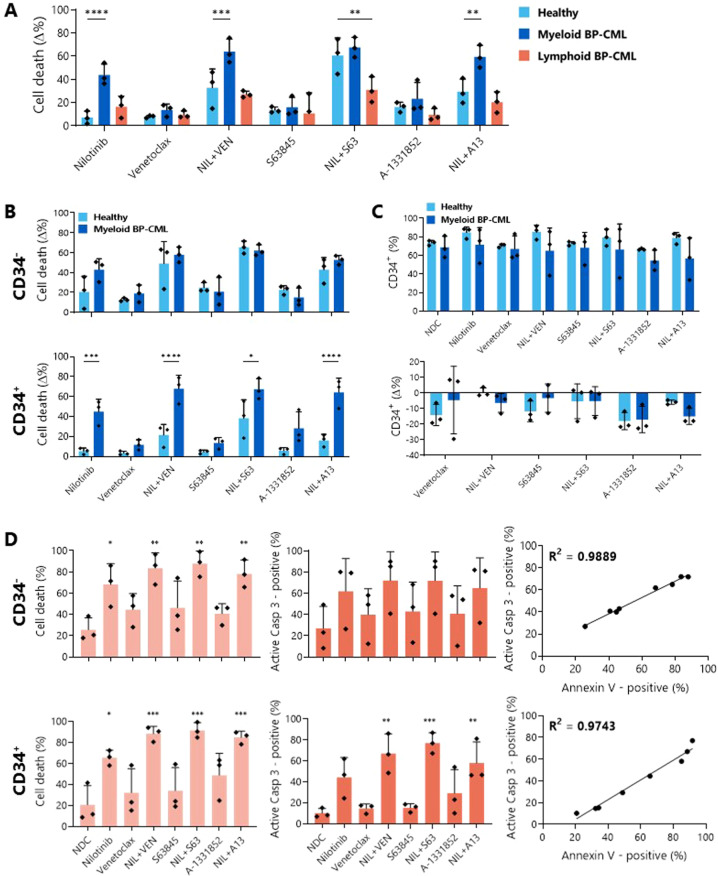
BH3 mimetics enhance the apoptotic effect of nilotinib in myeloid BP-CML patients and discriminately kill primitive CD34^+^ BP-CML cells. Three healthy, three myeloid BP-CML, and three lymphoid BP-CML patient samples were treated with 5 µM nilotinib and/or 1 µM BH3 mimetic as indicated for 72 h before Annexin V/DAPI staining in the bulk cell population (**A**), and in the CD34^-^ and CD34^+^ fractions of three healthy and three myeloid BP-CML samples (**B**). **C** After the same treatment, three healthy and three myeloid BP-CML samples were tested for CD34-positivity, expressed as a percentage of the whole cell population (top) and as a change compared with nilotinib alone (bottom; **C**). **A**, **B** and **C** analyzed by two-way ANOVA. **D** Apoptosis was confirmed by staining for Annexin V/DAPI (light orange) and active caspase-3 (dark orange) in CD34^-^ and CD34^+^ myeloid BP-CML cells after 72 h treatment with 5 µM nilotinib and/or 1 µM BH3 mimetics and correlation between both was determined, *N* = 3, analyzed by one-way ANOVA. NIL nilotinib, VEN venetoclax, S63 S63845, A13 A-1331852. *p ≤ 0.05, **p ≤ 0.01, ***p ≤ 0.001, ****p ≤ 0.0001. Data are represented as mean ± SD.

Next, we tested patient samples, with three healthy donor, three myeloid BP-CML (as used previously), and three lymphoid BP-CML samples treated with 5 µM nilotinib and/or 1 µM BH3 mimetics and Annexin V/DAPI staining was measured after 72 h (Fig. 8A). Nilotinib has a number of other kinase targets in addition to BCR::ABL1, including Abelson 1, KIT and platelet derived growth factor receptor [42], which results in apoptosis of healthy cells as well as BP-CML cells, although to a lesser degree.

Analysis of the bulk cell populations showed a significant increase in cell death for myeloid BP-CML when compared to healthy donor samples for nilotinib alone (healthy: 7.08 ± 5.55%; myeloid BP-CML: 43.9 ± 9.26%; *p* < 0.0001), and in combination with VEN (healthy: 32.9 ± 16.17%; myeloid BP-CML: 64.05 ± 10.71%; *p* = 0.0009) and A-1331852 (healthy: 29.57 ± 10.76%; myeloid BP-CML: 59.53 ± 9.77%; *p* = 0.0014). Further, while the addition of S63845 or A-1331852 to nilotinib treatment increased the amount of cell death observed over nilotinib alone in healthy cells, this effect was greater in the myeloid BP-CML cells (nilotinib+VEN: +20.15% cell death over nilotinib alone, *p* = 0.0054; nilotinib+S63: +23.60%, *p* = 0.0069; nilotinib+A13: +15.63%; *p* = 0.0017), indicating that co-inhibition of BCL-2 prosurvival proteins, particularly BCL-2 or MCL-1, with nilotinib could be a promising therapeutic strategy (Fig. 8A, Supplementary Table 6).

Identification of the more primitive CD34^+^ population within the myeloid BP-CML samples highlighted the discriminatory effect of nilotinib alone (healthy: 5.28 ± 3.13% cell death; BP-CML: 44.95 ± 12.44%; *p* = 0.0005), with VEN (healthy: 21.43 ± 10.8%; BP-CML: 67.79 ± 13.63%; *p* < 0.0001), with S63845 (healthy: 38.25 ± 18.58%; BP-CML: 67.35 ± 10.56%; *p* = 0.013), and with A-1331852 (healthy: 16.15 ± 6.02%; BP-CML: 64.27 ± 14.26%; *p* < 0.0001) compared with the healthy donor samples, while these distinctions could not be made within the CD34^−^ population (Fig. 8B). Crucially, treatment of the myeloid BP-CML samples with nilotinib and either VEN (+22.84% cell death; *p* = 0.0032) or S63845 (+19.32%; *p* = 0.0079) significantly increased apoptosis over nilotinib alone, while this was not the case for the healthy samples (Fig. 8B, Supplementary Table 7). However, this did not translate into a statistically significant reduction in the primitive CD34^+^ fraction in the myeloid BP-CML samples compared to healthy donor samples (Fig. 8C).

Apoptotic cell death was confirmed in the patient samples through correlation between PS presentation and caspase-3 activation (Fig. 8D). These results suggest that co-inhibition of BCR::ABL1 with BCL-2 prosurvival proteins, particularly BCL-2 and BCL-xL, kills myeloid BP-CML cells to a greater extent than their healthy counterparts.

## Discussion

This study aimed to investigate the potency of BH3 mimetics in BP-CML through cell line and patient sample testing, and our results suggest a functional dependence of BP-CML cells on MCL-1 and BCL-xL, with significant degrees of apoptosis induced, particularly after dual-inhibition.

Interestingly, combining BH3 mimetics with the TKI nilotinib was not a successful strategy for inducing apoptosis in lymphoid BP-CML samples. This was surprising, as the lymphoid BP-CML cell lines BV173 and CML-T1 were highly sensitive to all BH3 mimetics tested. As previous TKI exposure is likely to affect BH3 mimetic sensitivity, it would also be worth investigating whether other TKIs in combination with BH3 mimetics may be a potent therapeutic option in lymphoid BP-CML. Dual targeting of prosurvival BCL-2 proteins has also been shown to be effective in Ph+ ALL [43, 44], and is worth further investigation in lymphoid BP-CML.

Despite this, the effects of these drug combinations on reducing cell counts and increasing cell death within BP-CML cell lines and, more importantly, within the primitive CD34^+^ population of myeloid BP-CML patient samples, to a greater degree than in healthy samples, provides a strong rationale for further clinical investigation.

The diverse genetic landscape of BP-CML, both intrinsic to the disease and induced by TKI exposure, is well-documented [10] and likely to contribute to the varying responses to therapy observed. Methods to help predict response may therefore be of therapeutic benefit and could include techniques such as BH3 profiling [45] in a step towards a more targeted precision medicine approach.

The potential therapeutic benefit of co-inhibiting BCL-2 family proteins with BCR::ABL1 has been demonstrated here, and the data merit further investigation, both in the context of longer-term cell assays and in vivo murine experiments, to determine whether there is scope for translation of these therapies to clinical trials. An additional weapon in the therapeutic arsenal against BP-CML is urgently needed and BH3 mimetics could represent a valuable avenue for further exploration.

## Materials and methods

### Cell culture and drugs

K562, KCL-22, BV173, and CML-T1 cells were cultured in RPMI 1640 media with 1% penicillin-streptomycin, 1 mM l-glutamine and either 10% (K562, KCL-22, CML-T1) or 20% (BV173) fetal bovine serum (ThermoFisher Scientific, Paisley, UK) and incubated at 37°C with 5% CO_2_ in a humidified atmosphere. KCL-22 T315I [37] and KCL-22 PonRes [38] cell lines were previously generated through continuous sub-lethal exposure to TKI (nilotinib and ponatinib, respectively) and were maintained as the KCL-22 parental cells, with the addition of 100 nM ponatinib for KCL-22 PonRes cells. See Supplementary Table 1 for cell line details and suppliers.

Patient samples were obtained with written informed consent in accordance with the Declaration of Helsinki and ethical approval from the Greater Glasgow and Clyde NHS Trust Research Ethics Committee. Primary samples were isolated from leukapheresis or peripheral blood samples and purified for CD34^+^ cells. Primary cells were cultured in serum-free media (Iscove’s Modified Dulbecco’s Medium (ThermoFisher Scientific, cat. 12440053), BIT 9500 Serum Substitute (diluted 1 in 5, StemCell Technologies, Grenoble, France, cat. 09500), l-glutamine (2 mM, ThermoFisher, cat. 25030-024), Penicillin-Streptomycin (100 U/mL, ThermoFisher, cat. 15140-122), and 2 Mercaptoethanol (0.1 mM, ThermoFisher, cat. 31350-010)) containing hIL-3, hIL-6, G-CSF (all at 20 ng/mL), SCF, hFlt3 (at 100 ng/mL; StemCell Technologies), and hIL-7 (20 ng/mL; PeproTech, London, UK), overnight post-thaw, followed by culture in these growth factors at a 1:100 dilution. See Supplementary Table 2 for patient sample details.

Viable cell counts were determined by Trypan Blue (ThermoFisher Scientific, cat. 15250061) exclusion using a hemocytometer. Stock solutions of drugs (Stratech Scientific Ltd, Ely, UK) were reconstituted in sterile DMSO and aliquots stored at −20 °C for short-term use and −80 °C for long-term storage.

### Cell viability assays

Cell viability to determine the half maximal inhibitory concentration (IC50) values and synergistic action of test drugs was measured by resazurin reduction assay as previously described [46]. Briefly, test drugs were diluted serially in 96-well plates and cells were seeded at 2.5 × 10^4^ cells/mL. Plates were incubated at 37 °C in 5% CO_2_ for 72 h (h), after which resazurin solution (Sigma-Aldrich, Dorset, UK, cat. 62758-13-8) was added; plates were then incubated for a further 4 h. A SpectraMax M5 Plate Reader (Molecular Devices, California, USA) was used for measuring fluorescence. IC50 values were determined using GraphPad 8 (GraphPad, California, USA) and combination indices (CI) were calculated using CompuSyn (ComboSyn Inc., New Jersey, USA) software according to the Chou-Talalay method [47]. A CI of ‘>1’ indicates an antagonistic, ‘1’ additive and ‘<1’ synergistic interaction.

### Flow cytometry

Apoptosis was determined by Annexin V-FITC/DAPI, and by active caspase-3-PE intracellular staining. For Annexin/DAPI staining, cell death was defined as 100% - [DAPI-negative, Annexin V-negative cells %]. For caspase-3 staining, cells were pre-washed with PBS, fixed with 4% paraformaldehyde and permeabilized with ice-cold 90% methanol. Patient samples were co-stained with anti-CD34-APC.

Where indicated, cells were pre-treated with 10 µM Q-VD-OPh (Stratech Scientific; cat. S7311) before treatment with drugs of interest and subsequent staining with Annexin V/DAPI.

Intracellular staining with anti-BCL-2-V450, anti-MCL-1-AlexaFluor®−647, and anti-BCL-xL-AlexaFluor®−488 was carried out on fixed and permeabilized cells and normalized to fluorescence minus one controls after confirming minimal background staining with isotype controls.

Staining reagents used were Annexin V – FITC (BD Biosciences, Oxford, UK, cat. 556419), DAPI (BD Biosciences, cat. 564907), Active Caspase-3 – PE (BD Biosciences, cat. 51-68655X), CD34 – APC (BD Biosciences, cat. 345804), Mouse Anti-Human BCL-2 – V450 (BD Biosciences, cat. 560637), MCL-1 (D2W9E) Rabbit mAb – AlexaFluor® 647 (Cell Signaling Technology, Hitchin, UK, cat. 78471), BCL-xL (54H6) Rabbit mAb – AlexaFluor® 488 (Cell Signaling Technology, cat. 2767), Mouse IgG1, K Isotype Control – V450 (BD Biosciences, cat. 560373), Rabbit (DA1E) mAb IgG XP® Isotype Control – AlexaFluor® 647 (Cell Signaling Technology, cat. 2985), and Rabbit (DA1E) mAb IgG XP® Isotype Control – AlexaFluor® 488 (Cell Signaling Technology, cat. 2975).

Data were captured using a BD FACSCanto™ II (BD Biosciences) and analyses were carried out using FlowJo v10 (FlowJo LLC, Oregon, USA).

### Polymerase chain reaction

After drug treatment, cells were harvested and lysed, and RNA was prepared using a Qiagen RNeasy Mini kit (Qiagen, Crawley, UK, cat. 74104) according to manufacturer’s directions. RNA concentration and purity was measured using a NanoDrop™ Spectrophotometer (NanoDrop Technologies, Wilmington, USA). 500 ng RNA per condition was converted to cDNA using the QuantiTect Reverse Transcription kit (Qiagen, cat. 250311) according to manufacturer’s instructions. All samples were run in duplicate for each target gene. Final primer concentrations were 0.4 µM, with 10 ng cDNA used per reaction. Cycles were run using a Biomark™ Real-Time PCR Analyser (ThermoFisher Scientific) and DNA content measured using PowerTrack SYBR™ Green (ThermoFisher Scientific, cat. 46012). See Supplementary Table 3 for primer sequences and cycle details.

### Western blots

Cells were lysed in the presence of cOmplete™ ULTRA Protease Inhibitor (Sigma-Aldrich, Dorset, UK, cat. 5892791001) and PhosSTOP™ Phosphatase Inhibitor (Sigma-Aldrich, cat. 4906845001), with protein quantified by Bradford detection assay using Quick Start™ Bradford Dye Reagent (BIO-RAD, Hertfordshire, UK, cat. 5000205) as previously described [48]. Protein extracts were prepared using NuPAGE™ LDS Sample Buffer (ThermoFisher, cat. NP0007) and NuPAGE™ Sample Reducing Agent (ThermoFisher, cat. NP0009) and electrophoresis was carried out using NuPAGE™ 4–12% Bis-Tris Gels (ThermoFisher, cat. NP0323) in Bolt™ MES SDS Running Buffer (ThermoFisher, cat. B0002) containing NuPAGE™ Antioxidant (ThermoFisher, cat. NP0005). Protein transfer was carried out in NuPAGE™ Transfer Buffer (ThermoFisher, cat. NP0006-1) onto Amersham™ Protran® Nitrocellulose Membranes (Sigma-Aldrich, cat. GE10600002). Membranes were stained with BCL2 (124) Mouse Ab (Cell Signaling Technology, cat. 15071S), MCL-1 Rabbit Ab (Cell Signaling Technology, cat. 4572S), BCL-xL (54H6) (Cell Signaling Technology, cat. 2764S), or SH-PTP2 (B-1) (Santa Cruz Biotechnology, Texas, USA, cat. sc-7384) and visualized using an Odyssey™ Fc Imaging System (LI-COR Biosciences, Cambridge, UK). Densitometry was performed using Image Studio and ratio of BCL2/MCL-1/BCL-xL:SHPTP2 calculated and normalized to parental KCL-22 cells.

### Colony-forming unit assays

Post-treatment, primary cells were thoroughly washed of drug and 3 × 10^3^ live cells were plated in 1.1 mL pre-thawed MethoCult™ H4434 Classic (StemCell Technologies; cat. 04434) containing 20 ng/mL hIL-7 (PeproTech; cat. 200-07) in 35 mm × 10 mm dishes. Colonies were maintained at 37 °C with 5% CO_2_. Colonies were classified and counted after at least ten days in culture.

### Statistical analyses

All statistical analyses were carried out using GraphPad Prism 8. Error bars show standard deviation (SD) with significance denoted as follows: *p ≤ 0.05; **p ≤ 0.01; ***p ≤ 0.001; ****p ≤ 0.0001. An ordinary one-way analysis of variance (ANOVA) was used for comparison of more than two groups, and a two-way ANOVA was used for multiple comparisons. For correlation between Annexin V/DAPI and active caspase-3, linear regression was used.

## Supplementary information

Supplementary Table 7

Supplementary Legends

Supplementary Figure 1

Supplementary Figure 2

Supplementary Table 1

Supplementary Table 2

Supplementary Table 3

Supplementary Table 4

Supplementary Table 5

Supplementary Table 6

Author agreement for additional authors

## Data Availability

All data generated or analyzed during this study are included in this published article [and its supplementary information files].
