# Supplementary material for: BH3 mimetics in combination with nilotinib or ponatinib represent a promising therapeutic strategy in blast phase chronic myeloid leukemia
Source: Cell Death Discov. 2022 Nov 15;8:457. doi: 10.1038/s41420-022-01211-1 (PMC9666353; doi:10.1038/s41420-022-01211-1)
Supplement: Supplementary file 2 — Supplementary Legends [file 41420_2022_1211_MOESM2_ESM.docx]

**Supplementary Figure 1**

Minimal off-target staining in protein level analysis by flow cytometry was confirmed with isotype controls. Intracellular staining for BCL-2, MCL-1, and BCL-xL and comparative staining with isotype controls in K562 cells was tested at increasing concentrations. AF647 = Alexa Fluor® 647, AF488 = Alexa Fluor® 488.

**Supplementary Figure 2**

Full, uncropped Western blots of KCL22 PTL, T315I, and PonRes cell lines, stained for BCL-2, MCL-1, BCL-xL, and the housekeeping protein SHPTP2. N=3.

**Supplementary Table 1**

BP-CML cell lines used with disease origins, patient details, and supplier information provided.

**Supplementary Table 2**

Healthy and BP-CML patient sample information and disease type.

**Supplementary Table 3**

Primer targets with forward and reverse 5’ to 3’ sequences and associated ENSEMBL identifiers. PCR cycle information with number of cycles, temperature, length of stage, and purpose of each stage given.

**Supplementary Table 4**

Significant differences between treatment conditions outlined in **Figure 3A** after one-way ANOVA analysis with means and p values given. Significant differences are highlighted in bold.

**Supplementary Table 5**

Significant differences between treatment conditions outlined in **Figure 3B** after one-way ANOVA analysis with means and p values given. Significant differences are highlighted in bold.

**Supplementary Table 6**

Significant differences between nilotinib alone and in combination with BH3 mimetics as outlined in **Figure 7A** after one-way ANOVA analysis with means and p values given. Significant differences are highlighted in bold.

**Supplementary Table 7**

Significant differences between nilotinib alone and in combination with BH3 mimetics as outlined in **Figure 7B** (bottom graph) after one-way ANOVA analysis with means and p values given. Significant differences are highlighted in bold.
