# Supplementary figures and images for: BH3 mimetics in combination with nilotinib or ponatinib represent a promising therapeutic strategy in blast phase chronic myeloid leukemia
Source: Cell Death Discov. 2022 Nov 15;8:457. doi: 10.1038/s41420-022-01211-1 (PMC9666353; doi:10.1038/s41420-022-01211-1)

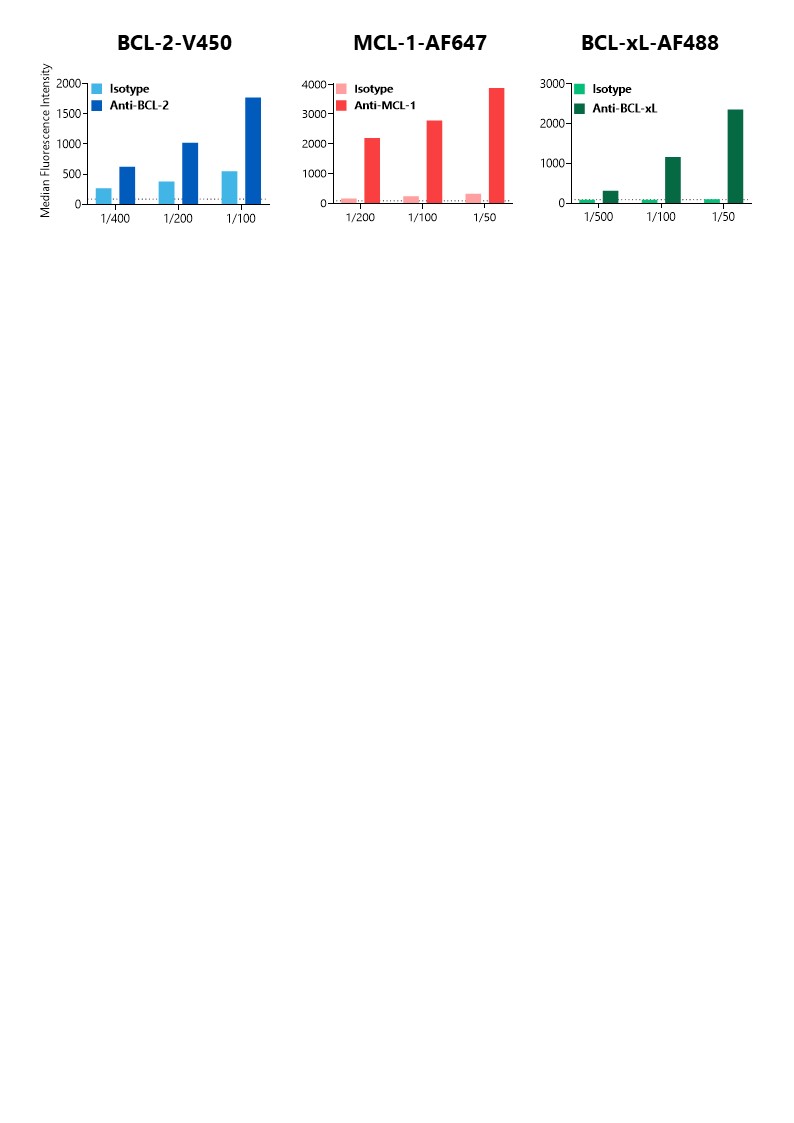

Supplement: Supplementary file 3 — Supplementary Figure 1 [file 41420_2022_1211_MOESM3_ESM.jpg]

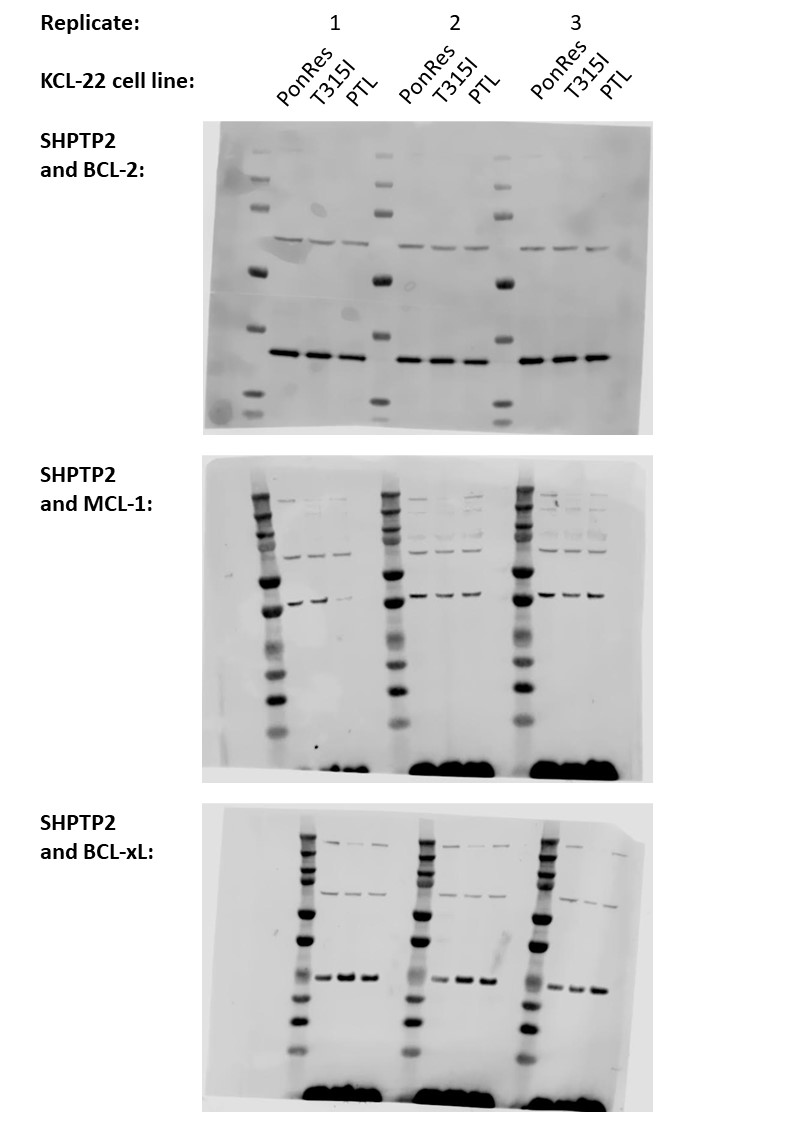

Supplement: Supplementary file 4 — Supplementary Figure 2 [file 41420_2022_1211_MOESM4_ESM.jpg]
